# Non-Contact Reflection-Coefficient Measurement of Automotive Transmission Fluid Degradation with a Hexagonal Patch Array Antenna: Measurement Model, Repeatability and Uncertainty Considerations †

**DOI:** 10.3390/s26206462

**Published:** 2026-10-13

**Authors:** Mehmet Duman

**Affiliations:** Department of Electrical and Electronics Engineering, Faculty of Engineering, Düzce University, 81620 Duzce, Türkiye; mehmetduman@duzce.edu.tr or mehmetduman@ieee.org

**Keywords:** RF-based fluid sensing, dielectric characterization of lubricants, non-contact oil condition monitoring, reflection-coefficient measurement, measurement uncertainty, repeatability, automotive transmission fluid degradation, hexagonal patch array antenna

## Abstract

**Highlights:**

**What are the main findings?**

- For the single fresh/degraded ATF sample pair examined in this study, degradation shifts the fluid-loaded resonance notch upward by ≈20 MHz and deepens it by ≈14.4 dB. Analysis of the complex reflection coefficient shows that both effects arise from one mechanism: the reactive part of the loaded aperture impedance collapses from +3.7 Ω to +0.4 Ω, while the resistive part is essentially unchanged.
- Across ten repeated acquisitions of these two specimens, the separation is ≥7.7 dB in the worst case, against a session drift of 0.31 dB measured by bracketing the degraded block between fresh-fluid acquisitions. The repeats characterise measurement repeatability on two specimens and do not characterise fresh and degraded ATF as fluid classes.

**What are the implications of the main findings?**

- Non-contact, low-cost reflection-coefficient measurement with a patch array antenna is a viable candidate for condition-based ATF maintenance without penetrating or contaminating the fluid.
- The explicit measurement model, repeatability data and uncertainty considerations provide a metrological baseline for future calibrated, multi-point implementations.

**Abstract:**

This study formulates and experimentally characterizes a non-contact reflection-coefficient (S_11_) measurement method for assessing the degradation state of automotive transmission fluid (ATF). The measurand is the reflection coefficient, at the SMA (SubMiniature version A) reference plane, of a 4 × 4 hexagonal patch array antenna whose near field is loaded by a steel-backed measurement cell containing a fluid layer of approximately 9–10 mm; fluid condition is read from the frequency and level of the fluid-loaded resonance notch. The antenna was characterized by full-wave simulation (resonance at 4.852 GHz) and by SOLT (short-open-load-thru)-calibrated bench measurements (4.725 GHz, −24.32 dB), with the far-field pattern measured in an anechoic chamber. Fluid spectra were acquired using a SOLT-calibrated LibreVNA (2–6 GHz, 4 MHz step) within a single session under one calibration in a bracketed sequence in which the fresh fluid was measured both before and after the degraded fluid. Repeated acquisitions (n = 6 fresh; n = 4 degraded, ~200,000 km of service) yield a fresh-fluid notch of −28.6 ± 0.6 dB at 4.755 GHz and a degraded-fluid notch of −43.0 ± 4.3 dB at 4.775 GHz. Decomposition of the measured complex reflection coefficient shows that the resistive part of the loaded impedance at the notch is essentially unchanged (49.9 → 50.5 Ω) whereas the reactive part collapses (+3.7 → +0.4 Ω); the upward frequency shift and the deepening are therefore two consequences of a single reactive mechanism rather than two independent signatures. The two specimens were separated at *p* = 0.0095 (level) and *p* = 0.0080 (frequency) by the Mann–Whitney U test against a bracketed session drift of −1.3 MHz and +0.31 dB. Type A repeatability results and Type B considerations are reported following the GUM/VIM. These results establish a metrologically characterized, low-cost, non-invasive proof of concept for ATF condition monitoring, validated here on a single fresh/degraded sample pair.

## 1. Introduction

Reliable condition monitoring of automotive lubricating fluids is essential for ensuring drivetrain longevity, minimizing unscheduled maintenance costs, and reducing the environmental burden associated with premature fluid disposal [1,2]. Transmission fluids undergo progressive degradation during service through several concurrent mechanisms, including thermal oxidation, shear-induced viscosity loss, particulate contamination from metallic wear debris, and chemical additive depletion [3,4,5,6]. These processes collectively alter the physicochemical and dielectric properties of the fluid in ways that can be correlated with its remaining useful life. Conventional monitoring strategies rely on fixed mileage- or time-based replacement schedules that do not account for actual fluid condition; such approaches may lead either to premature disposal—generating unnecessary hazardous waste—or to excessively prolonged use that risks mechanical component damage. Consequently, there is a growing demand for real-time or near-real-time sensing technologies capable of objectively assessing fluid condition and supporting data-driven maintenance decisions in automotive systems.

A variety of sensing modalities have been investigated for lubricant condition assessment. Electrochemical sensors employing two-electrode configurations have been utilized for in situ oil analysis in passenger vehicles [7], while cost-effective permittivity-based sensors have been proposed for monitoring changes in the complex dielectric properties of lubricants [8]. Systems combining viscosity, permittivity, and wear-debris measurements have been developed for online degradation monitoring in industrial equipment [2], Internet of Things (IoT)-enabled platforms that correlate electrical signals with engine oil quality indicators have also been reported [9], and online spectral analyzers have been investigated for continuous lubricant quality assessment [10]. Additionally, optical methods—including infrared absorption and fluorescence spectroscopy—have been applied to evaluate synthetic lubricant degradation in aerospace gearboxes [3,4]. Dielectric spectroscopy in particular has been shown to track engine-oil oxidation quantitatively, correlating with total acid number (TAN) and insoluble content [11], and combined water-content/conductivity/permittivity monitoring confirms that dielectric parameters increase systematically with oxidation time [12]; direct measurements of ATF dielectric properties are also available [13]. While these approaches offer valuable diagnostic capabilities, many require direct fluid contact, involve complex sample preparation, or demand expensive instrumentation, thereby limiting their suitability for routine in-vehicle deployment.

Radio frequency (RF) and microwave sensing techniques have emerged as a promising non-contact alternative for dielectric material characterization, exploiting changes in electromagnetic wave propagation, reflection, and resonance parameters to infer material properties without physical contact with the sample under test [14,15]. The breadth of the approach is reflected in recent surveys of microwave permittivity sensing, which map the principal resonant, transmission-line and reflection-based architectures together with the permittivity-extraction methods associated with each [16]. Within this framework, antenna-based sensors have attracted increasing research attention. A recent review of microstrip patch antenna-based passive sensors sets out how frequency shift, return-loss and impedance variation are exploited as sensing mechanisms in this class of device, and identifies the absence of standardised characterisation as a recurring weakness of the literature [17]. Zhu et al. [18] demonstrated a microfluidic microstrip patch antenna sensor for monitoring moisture content in lubricating oil via resonance frequency shifts. Rajendran et al. [19] proposed a self-complementary dipole antenna for liquid adulteration detection across various fluid types, although the study did not extend to automotive fluid applications. Jun et al. [20] explored an electromagnetic bandgap (EBG) structure combined with planar antenna elements as a liquid sensor, reporting measurable S_11_ changes across different liquid media, and Arif et al. [21] demonstrated an EBG-based sensor for dielectric characterization of liquids. Vafanejad [22] investigated microacoustic shear-mode resonators for lubricating oil quality assessment, focusing on density and viscosity measurements. Ikhmis et al. [23] developed a helical antenna sensor for liquid-type identification. More broadly, Shinde et al. [1] focused on integrated sensory systems for engine oil quality assessment. Several non-contact RF and microwave sensing approaches have also been reported: Dalgaç et al. [24] employed chiral metamaterial-based sensors to assess the quality of car lubrication oil at X-band; Baghelani et al. [25] proposed a highly sensitive planar microwave resonator for detecting water contamination in mineral oil at ppm-level sensitivity; Chuma et al. [26] developed a microwave metamaterial resonator integrated with neodymium magnets for detecting wear particles in oil; and Zhang et al. [27] demonstrated a submersible microwave sensor for edible oil quality analysis. Palandoken and Gocen [14] recently provided a comprehensive review of technological advances in microwave sensor designs for liquid material dielectric characterization, covering frequency-variation, phase-variation, and differential-mode sensor configurations. Wu et al. [15] proposed a microwave sensing system combining an interdigital-structure-based microstrip line with RF circuits for extracting complex permittivity of liquid samples, achieving a sensitivity improvement of 57.5–175.3% over prior planar resonator approaches. Within the last five years, microwave oil sensing has advanced along several directions directly relevant to the present work: microfluidic microwave sensors with annular microstrip patches for lubricating-oil quality inspection [28], additively manufactured X-band waveguide sensors for engine-oil quality monitoring [29], and dielectric cavity antenna sensors capable of discriminating fresh from service-aged engine oils [30]. Antenna-based diagnostics have also been extended beyond lubricants to insulating fluids, where a dielectric resonator antenna was used to track the condition of transformer insulation oil from its resonant response, demonstrating that the antenna-as-sensor principle transfers across oil types [31]. These recent studies, however, address engine or edible oils, operate in contact or immersion configurations, and report sensor responses without an accompanying repeatability or uncertainty characterization. A comparative summary of representative works is presented in Table 1.

Despite this growing body of work, two gaps remain. First, in terms of application, array-antenna-based reflection measurements have not been applied to automotive transmission fluids, as opposed to engine oils or generic liquids (Table 1). Second—and more importantly from a measurement-science perspective—prior antenna-based oil-sensing studies typically report a sensor response without formulating the underlying measurement problem: the measurand is left implicit, the measurement chain and its calibration are only partially specified, and repeatability or uncertainty information is absent. The present study addresses both gaps by formulating and experimentally characterizing a non-contact reflection-coefficient measurement method for ATF degradation assessment. The contributions are: (i) an explicit measurement model in which a steel-backed layered fluid cell (air gap/fluid layer/metallic backing) is interrogated through the reflection coefficient of a 4 × 4 hexagonal patch array antenna, with the measurand, the measurement chain, the reference planes and the dominant influence quantities identified (Section 3.1); (ii) a degradation signature comprising an upward shift of ≈20 MHz and a deepening of ≈14.4 dB of the fluid-loaded resonance notch, shown in Section 3.2 to arise from a single reactive mechanism rather than from two independent effects, quantified from repeated acquisitions (n = 6 fresh, n = 4 degraded) rather than from single traces; (iii) a repeatability (Type A) and instrument-related (Type B) uncertainty characterization of the method, including SOLT calibration at a defined reference plane verified with an independent calibration kit (Section 3.6); and (iv) transparent identification of the metrological limitations that separate this proof of concept from a calibrated, deployable measurement system. The disciplined use of metrological terminology throughout the paper follows the Guide to the Expression of Uncertainty in Measurement (GUM) [32] and the International Vocabulary of Metrology (VIM) [33].

## 2. Antenna Design, Simulation and Experimental Validation

### 2.1. Antenna Geometry and Design Rationale

The sensing antenna is a 4 × 4 planar array based on hexagonal microstrip patch elements. The hexagonal patch geometry was selected because its multi-sided shape supports additional higher-order resonant modes compared to rectangular or circular patches of equivalent area, resulting in a multi-resonant frequency response—a characteristic confirmed by the full-wave simulations in Section 2.2. Each radiating element consists of a regular hexagonal patch with a side length of 10 mm.

The antenna comprises 16 hexagonal microstrip patch elements arranged in a planar 4 × 4 grid (four columns of four elements). The excitation uses a hybrid corporate–series feed architecture: a 1-to-4 corporate power divider distributes the input among four column sub-arrays, and within each column the four elements are interconnected by a series feed. The structure therefore presents four independently fed sub-arrays and a 16-element radiating aperture. The designation 4 × 4 refers to the radiating aperture, which is the property that governs the sensing function: the near-field footprint of the 16-element array integrates the dielectric response over the fluid reservoir, and the aperture dimension (≈100 × 100 mm^2^) was deliberately matched to the reservoir footprint.

The operating frequency of the hexagonal element can be estimated from the cavity model of the equivalent circular patch [34]. A regular hexagon of side a has the same area as a circle of equivalent radius

a_eq_ = a·√(3√3/2π) ≈ 0.909 a (1)

which gives a_eq_ ≈ 9.09 mm for a = 10 mm. Accounting for the fringing fields through the standard effective-radius correction for a circular patch on a substrate of height h = 1.6 mm and ε_r_ = 4.3 [34] yields an effective radius a_e_ ≈ 9.55 mm, and the dominant-mode resonance follows as

f_r = 1.8412·c/(2π·a_e_·√ε_eff_) (2)

With ε_eff_ = 3.424 (Section 2.2), Equation (2) evaluates to ≈ 4.97 GHz. This closed-form estimate lies within ≈2.4% of the full-wave simulated resonance of 4.852 GHz, providing the physical design rationale for the chosen element size.

**Figure 1:**
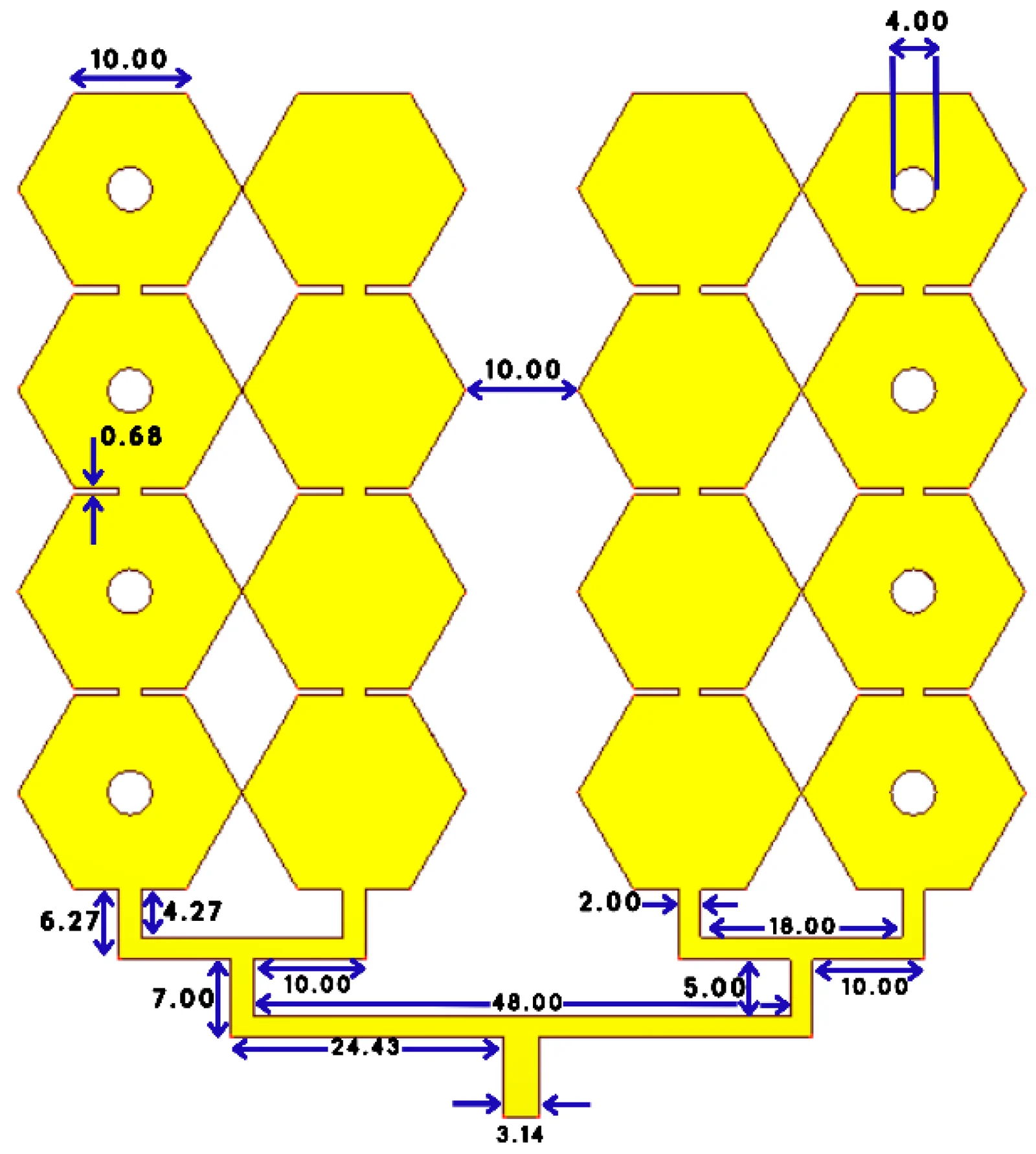
Layout and dimensional parameters of the proposed 4 × 4 hexagonal patch array antenna (all dimensions in mm). The 16 hexagonal elements are arranged in four columns of four. The 1-to-4 corporate divider at the bottom edge distributes the input among the four column sub-arrays; within each column the elements are interconnected by a series feed at an 18 mm pitch. The input line joins the 48 mm distribution line 24.43 mm from its left end, which is the feed asymmetry discussed in Section 2.4 and Section 2.5.

The corporate feed is designed to give nominally equal-amplitude, equal-phase excitation at each sub-array port. The element-to-element pitch within each column is 18 mm, taken from the fabricated layout of Figure 1. At the 4.852 GHz design frequency this corresponds to ≈0.29 λ_0_ in free space and ≈0.54 λ_g in the microstrip medium, where λ_g = λ_0_/√ε_eff = 33.4 mm for ε_eff = 3.424. Because the pitch is well below λ_0_, grating lobes are not excited. The electrical length of the line connecting successive elements is, however, the quantity that fixes the progressive phase of the series feed, and hence the E-plane beam direction examined in Section 2.5. All feed paths converge into a single 50 Ω SMA input port. As dimensioned in Figure 1, the feed network is not exactly mirror-symmetric: the input line joins the 48 mm distribution line 24.43 mm from its left end, and the left- and right-hand sub-feed sections have unequal segment lengths (e.g., 7.00 mm versus 5.00 mm junction stubs). This small, deterministic path-length imbalance between the column pairs is revisited in Section 2.4. The geometry and dimensional parameters are presented in Figure 1.

### 2.2. Full-Wave Electromagnetic Simulation

**Figure 2:**
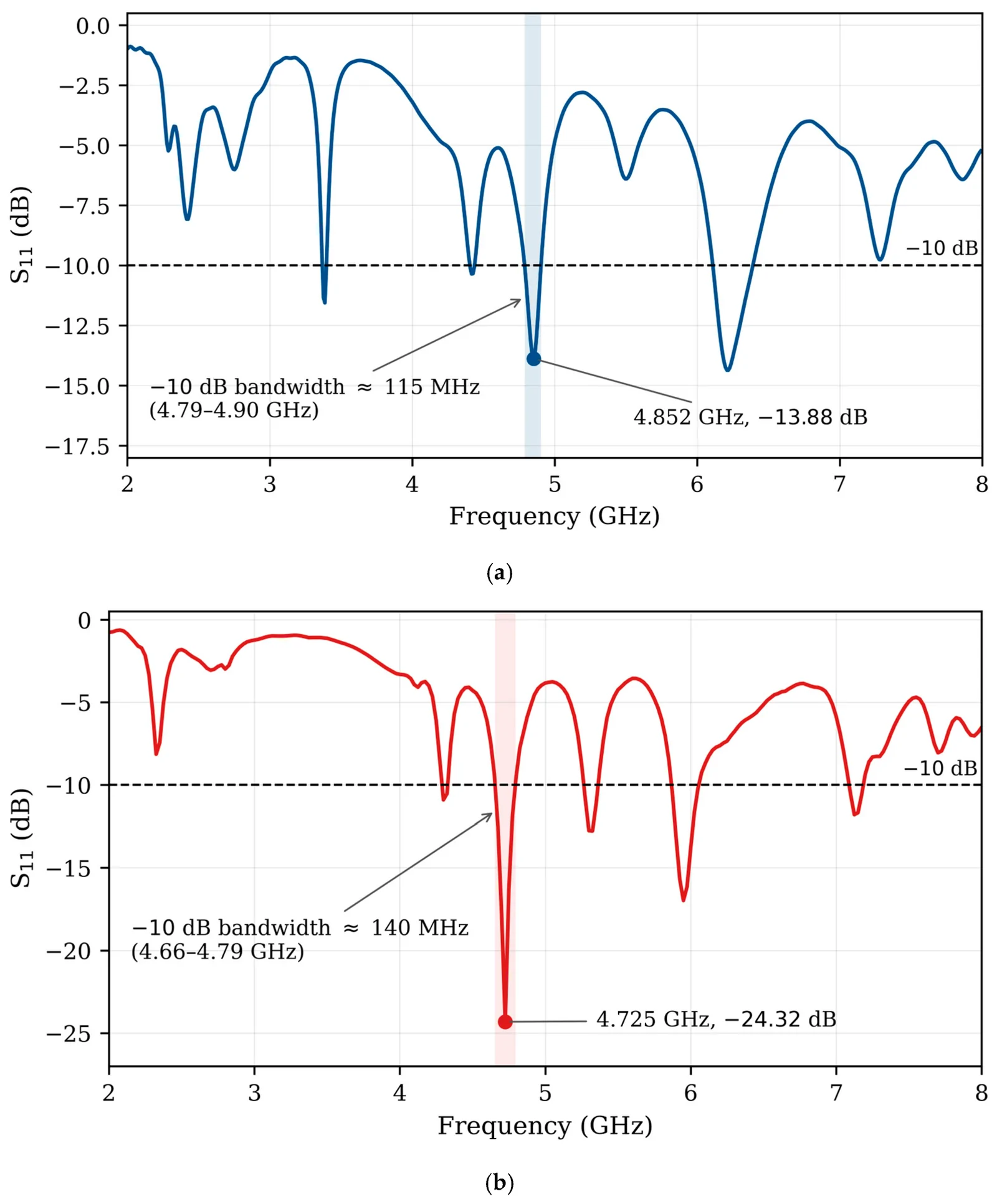

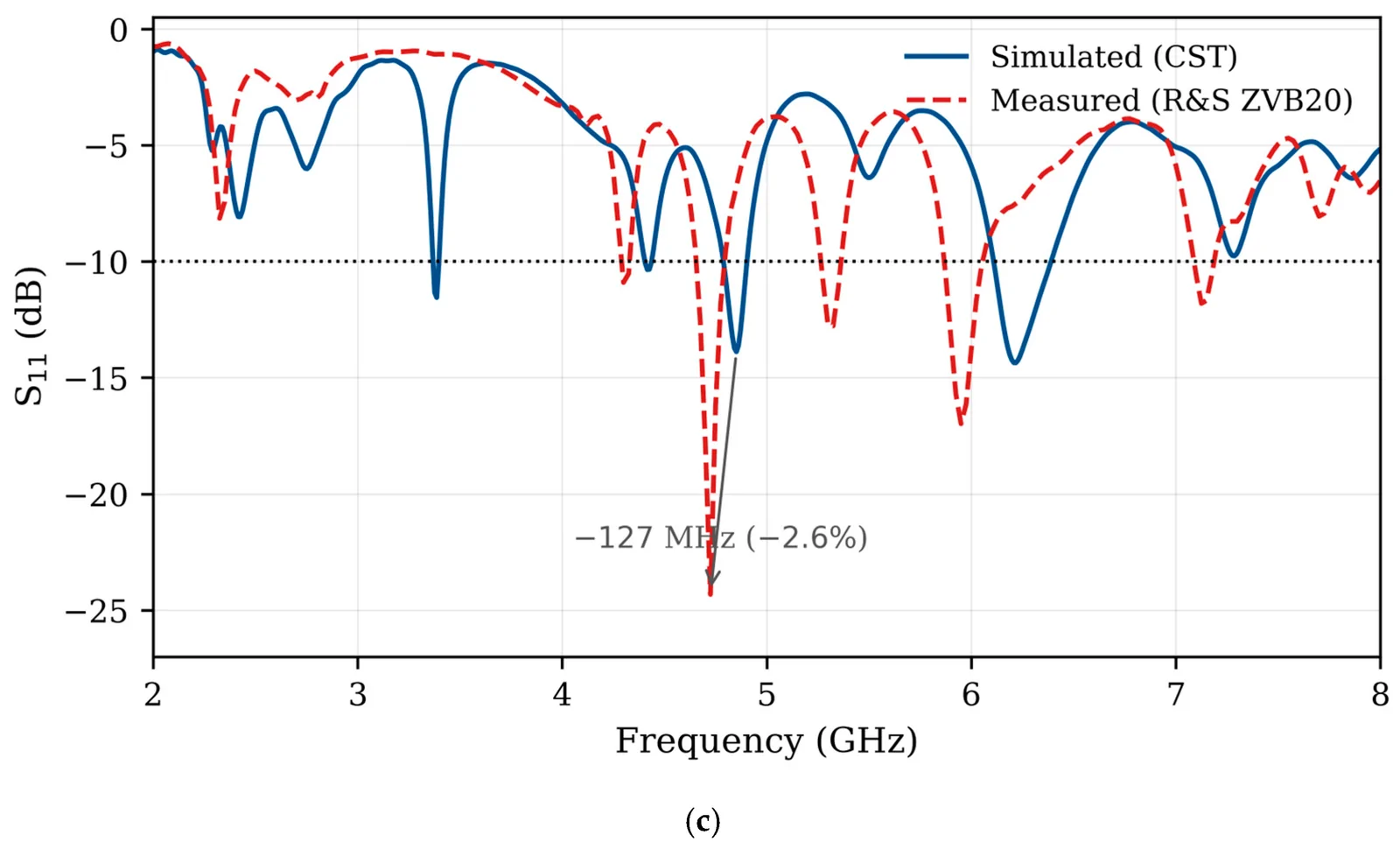
(**a**) Simulated S_11_ response (CST; the shaded band marks the ≈115 MHz −10 dB bandwidth of the 4.852 GHz resonance); (**b**) measured S_11_ response (R&S ZVB20, SOLT-calibrated at the SMA reference plane; 25 MHz frequency step; the shaded band marks the ≈140 MHz −10 dB bandwidth); (**c**) overlay of simulated and measured S_11_ responses (the arrow marks the −127 MHz/−2.6% downshift of the main resonance).

Full-wave electromagnetic simulations were performed using the CST (Computer Simulation Technology) Microwave Studio time-domain solver. The antenna was modeled on an FR4 substrate modeled on an FR4 (flame-retardant 4) substrate with εr = 4.3, tan δ = 0.025, and thickness = 1.6 mm. The frequency-dependent effective dielectric constant, computed by CST’s built-in dispersion model and equal to 3.424 at the design frequency, is plotted in Figure 2 essentially constant across the band of interest and equal to 3.424 at the design frequency. Simulation results predicted a resonant frequency of 4.852 GHz with a minimum S11 of −13.88 dB, as shown in Figure 2a. The −10 dB impedance bandwidth of this resonance, computed from the exported simulation data, is ≈115 MHz (4.79–4.90 GHz). The structure is multi-resonant: additional sub-−10 dB intervals occur near 3.38 GHz (~20 MHz), 4.42 GHz (~30 MHz), and 6.12–6.39 GHz (~270 MHz). The markers close to the notch minimum indicate a local bandwidth measured a fixed number of decibels above the minimum, which is a different (and narrower) quantity than the −10 dB impedance bandwidth; both are distinguished explicitly here.

### 2.3. Fabrication and S_11_ Measurement

Following fabrication by standard photolithographic etching on FR4, the prototype antenna was characterized on the bench by direct connection to a Rohde & Schwarz ZVB20 vector network analyzer (VNA), USA. Prior to measurement, a full Short-Open-Load-Thru (SOLT) calibration was performed with the manufacturer’s calibration kit, transferring the calibration reference plane to the SMA connector of the antenna under test and thereby removing cable and connector insertion losses from the S_11_ measurement; the anechoic chamber was used for the far-field pattern measurements described in Section 2.5. The reported sweep covers 2–12 GHz with 401 points (25 MHz frequency step). The measured S_11_ response exhibited a resonant frequency of 4.725 GHz with a minimum S_11_ of −24.32 dB (Figure 2b); the measured −10 dB bandwidth of this notch is ≈140 MHz (4.66–4.79 GHz). Because the deep-notch region is narrower than the 25 MHz frequency step of this sweep, the sampled minimum value carries a grid-resolution contribution that is accounted for in Section 3.6. An independent 4–6 GHz sweep acquired in a separate measurement session located the same notch at 4.760 GHz with a minimum of ≈−40 dB, illustrating that the notch frequency is reproducible to within ≈35 MHz between sessions whereas the exact depth of a deep, well-matched notch is highly sensitive to cable routing and connector repeatability—an expected property of deep reflection minima. The overlay of simulated and measured S_11_ curves is shown in Figure 2c.

The measured resonant frequency shifted downward by approximately 127 MHz (2.6%) relative to the simulation, attributable to manufacturing tolerances in the photolithographic etching process, variations in the actual substrate permittivity from the nominal FR4 value, and parasitic effects of the SMA connector. Notably, the measured notch (−24.32 dB) is significantly deeper than the simulated value (−13.88 dB), indicating superior impedance matching in the fabricated prototype. Following the standard sign conventions, S_11_ expressed in decibels is negative for a matched antenna, while the return loss RL = −20·log10|Γ| is the corresponding positive quantity (e.g., RL = 24.32 dB at 4.725 GHz); throughout this paper the S_11_ (dB) convention is used consistently. The close agreement in spectral shape between simulated and measured S_11_ responses confirms the validity of the electromagnetic model.

### 2.4. Impedance and Near-Field Characteristics

The simulated input impedance at resonance is 48.5 Ω, in close proximity to the 50 Ω reference impedance. Since the voltage standing wave ratio carries the same information as the reflection coefficient, only S_11_ is reported in this paper; the corresponding matching quality is fully described by the S_11_ levels and −10 dB bandwidths given in Section 2.2 and Section 2.3.

**Figure 3:**
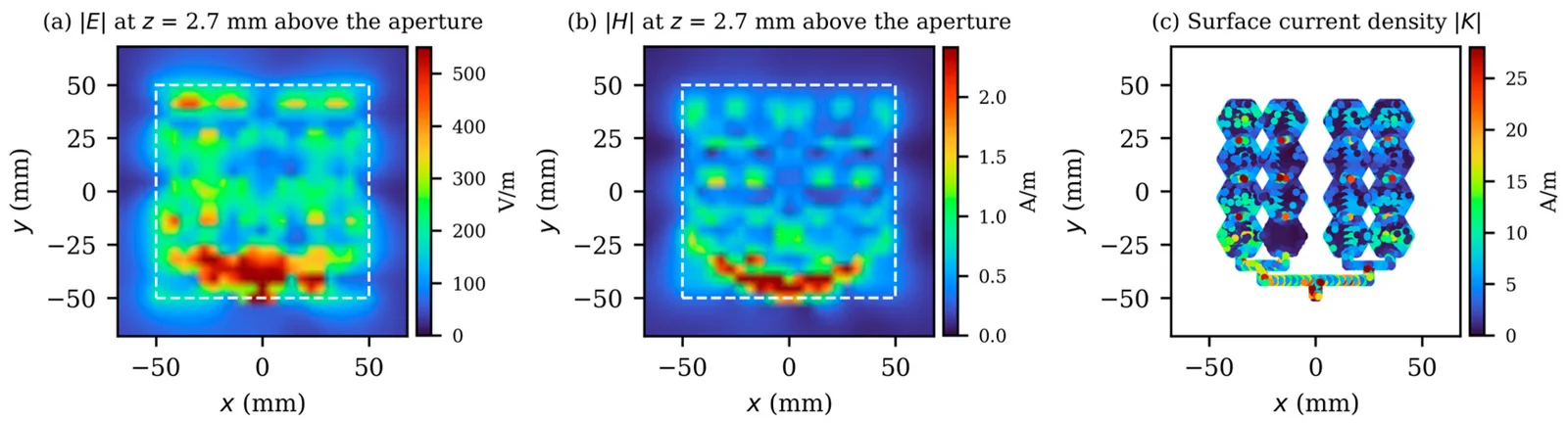
Simulated field distributions of the array at 4.725 GHz: (**a**) electric field magnitude |E| and (**b**) magnetic field magnitude |H|, both evaluated on a plane 2.7 mm above the radiating aperture, i.e., at approximately the height occupied by the fluid surface in the measurement configuration of Section 3; (**c**) surface current density |K| on the conductor layer. The dashed square in (**a**,**b**) marks the 100 × 100 mm^2^ board outline. The near field in (**a**,**b**) is distributed across the full aperture, which supports the aperture-averaging argument of Section 2.1, while (**c**) resolves the corporate–series feed network and the element excitation. The distributions are evaluated at 4.725 GHz, the measured resonance and the frequency at which the fluid measurements of Section 3 are performed; the corresponding distributions at the simulated resonance of 4.852 GHz are closely similar (the surface-current magnitudes correlate at r = 0.91) and are not reproduced separately.

The simulated E-field, H-field, and surface current distributions at the resonant frequency are presented in Figure 3. The field distributions show that all hexagonal patches contribute to the radiated field, with the strongest excitation at the central elements. Within each series-fed column sub-array, elements at the extremities receive slightly less excitation than those nearest the column input port—a well-known characteristic of series feed networks. The left–right asymmetry visible in the surface-current plot (Figure 3c) has two identified causes. First, the corporate feed network is not exactly mirror-symmetric:

Figure 3c is computed from the exported complex current components as the phasor magnitude |K| = (|Kx|^2^ + |Ky|^2^ + |Kz|^2^)^(1/2), which is independent of phase reference. The asymmetry visible in Figure 3c is therefore a genuine property of the excitation and is attributable to the feed-network asymmetry identified above. The same asymmetry accounts for the E-plane beam squint reported in Section 2.5, and both reinforce the recommendation that future designs adopt a fully symmetric corporate feed in both dimensions.

### 2.5. Far-Field Radiation Pattern

**Figure 4:**
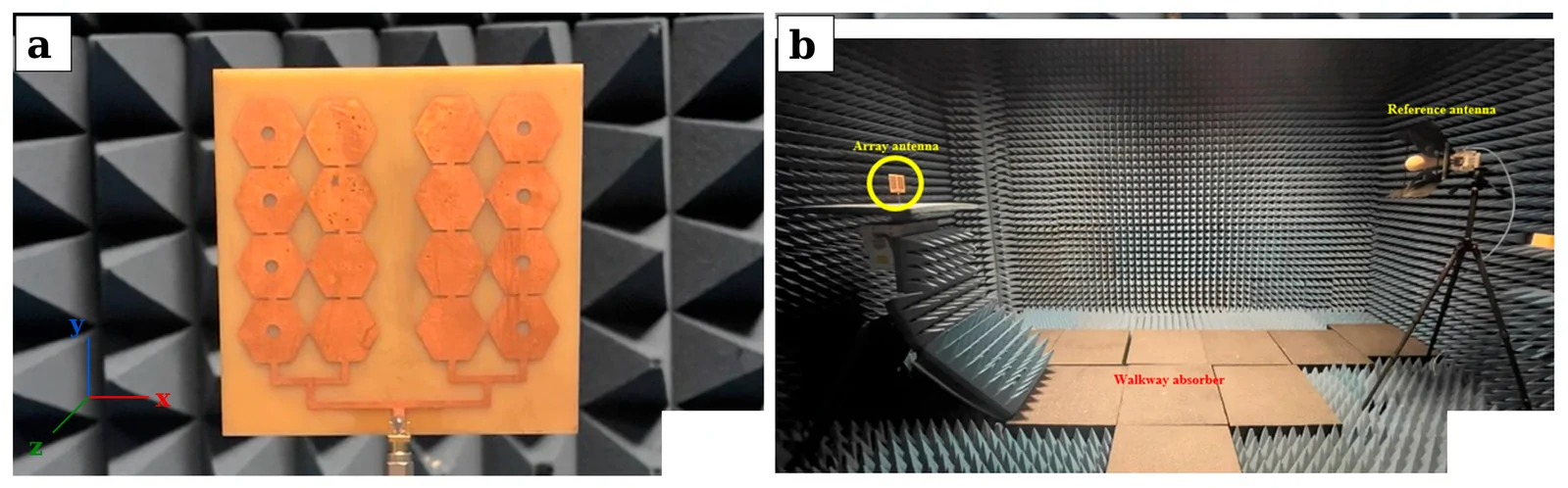
(**a**) Fabricated antenna under test (AUT) mounted on the rotational positioner [35]; (**b**) radiation pattern measurement configuration inside the anechoic chamber with the DE0530 broadband dual-ridge reference horn at a separation of 2.4 m.

**Figure 5:**
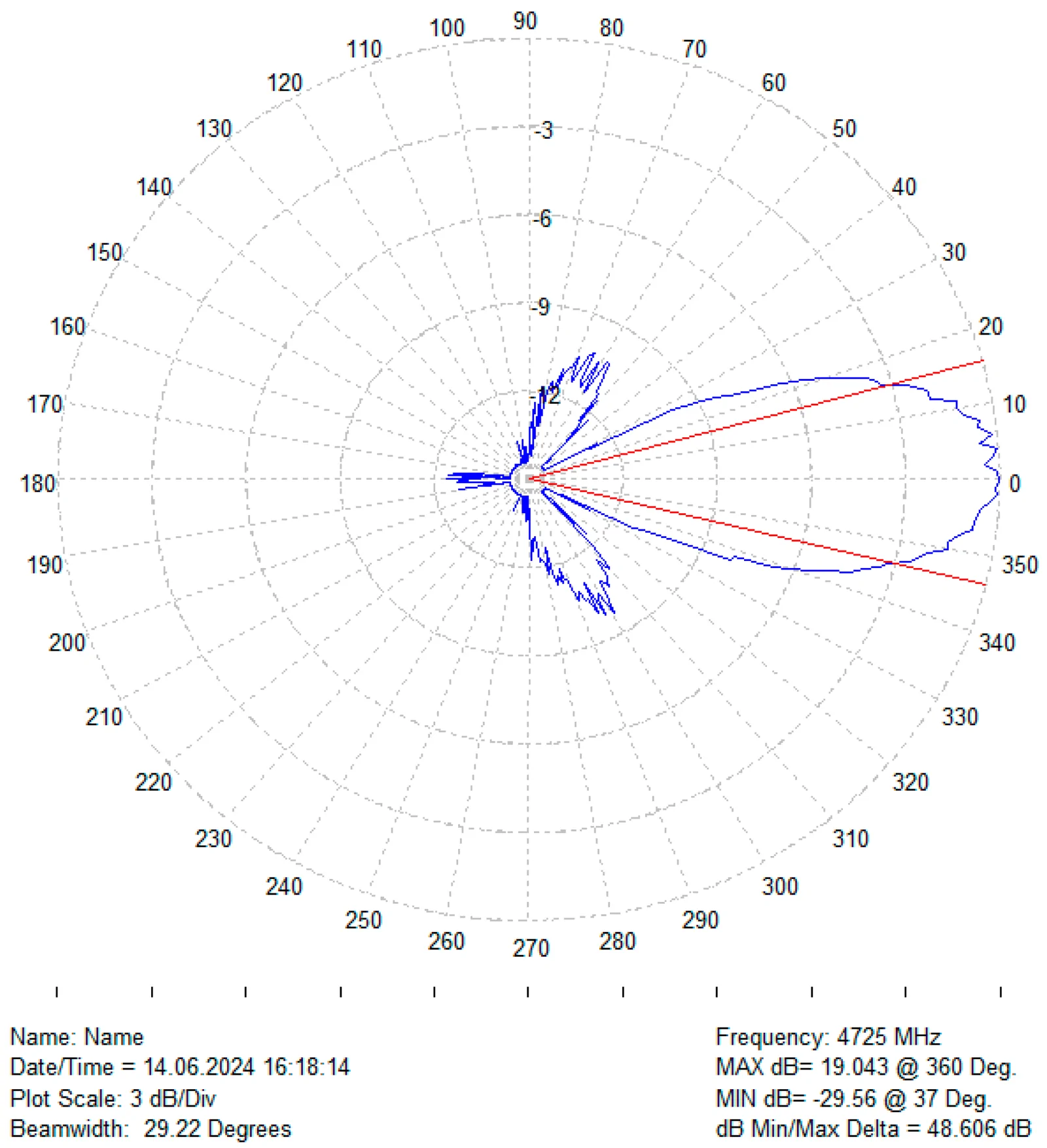
Measured far-field radiation pattern of the proposed 4 × 4 hexagonal patch array antenna at 4.725 GHz (relative received power, 3 dB/division; software level annotations are uncalibrated relative values, see Section 2.5). The measurement is the 360° azimuthal sweep of the positioner and therefore corresponds to the x–z plane of the antenna coordinate system defined in Figure 4; the two straight radial markers indicate the –3 dB half-power beamwidth (HPBW) of 29.22°.

The far-field radiation pattern of the fabricated antenna was characterized in an anechoic chamber facility using a Diamond Engineering DAMS positioner system with the accompanying Antenna Measurement Studio software, USA. The antenna under test (AUT) was mounted on a rotational positioner and measured against a broadband dual-ridge reference horn (Diamond Engineering DE0530, 0.5–30 GHz, USA) across a full 360° azimuthal sweep at 4.725 GHz. The AUT–horn separation was 2.4 m, comfortably satisfying the far-field criterion 2D^2^/λ ≈ 0.63 m for the D ≈ 0.14 m aperture at 4.725 GHz. Figure 4 depicts the AUT and the measurement configuration; Figure 5 presents the measured radiation pattern.

**Figure 6:**
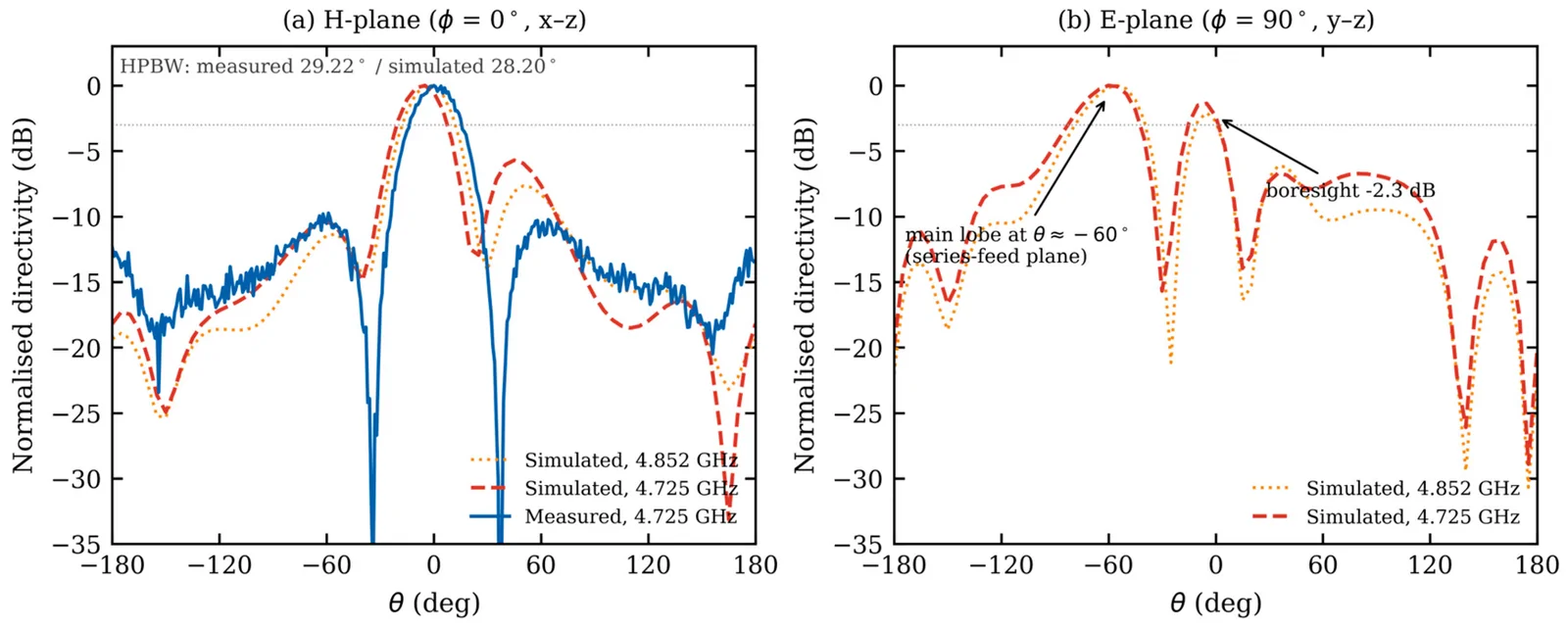
Simulated principal-plane radiation patterns of the array, normalised to the maximum of each curve: (**a**) H-plane (φ = 0°, the x–z plane of Figure 4), with the measured pattern of Figure 5 overlaid; (**b**) E-plane (φ = 90°, the y–z plane, which contains the series-fed columns). In the H-plane the simulated and measured patterns agree closely, with half-power beamwidths of 28.20° and 29.22° respectively. In the E-plane the main lobe is squinted to θ ≈ −60° and the boresight level is 2.3 dB below it, which is the expected behaviour of a series-fed column and is discussed in Section 2.5. No measurement is available in the E-plane.

Simulated patterns in both principal planes are presented in Figure 6 for comparison with the measurement. In the H-plane (φ = 0°, the x–z plane of Figure 4), which is the plane sampled by the azimuthal sweep of the positioner, the simulated and measured patterns agree closely: the simulated half-power beamwidth is 28.20° against the measured 29.22°, a difference of 3.5%, and the positions and levels of the first side lobes correspond. This agreement in the measured plane supports the validity of the electromagnetic model.

In the E-plane (φ = 90°, the y–z plane, which contains the series-fed columns) the simulation predicts a different behaviour that the present measurement campaign did not sample. The main lobe is not at boresight but is squinted to θ ≈ −60°, and the boresight level lies 2.3 dB below the lobe maximum. This is the expected behaviour of a series-fed linear array: the progressive phase between successive elements is fixed by the electrical length of the interconnecting line, and when that length departs from one guided wavelength at the operating frequency the array factor steers away from broadside. The effect appears only in the plane containing the series feed, which is consistent with the H-plane remaining centred on boresight, and with the feed-network asymmetry already identified in Section 2.4. It reinforces the recommendation made there that future designs adopt a fully symmetric corporate feed in both dimensions.

This squint does not affect the fluid measurement reported in Section 3. The sensing configuration operates with the radiating face within approximately 2 mm of the fluid surface, that is, in the reactive near field of the aperture, where the far-field beam direction is not the governing quantity; the relevant property is the near-field footprint of the 16-element aperture over the reservoir, as set out in Section 2.1. The far-field characterisation is reported here because it documents the fabricated antenna, not because the sensing mechanism depends on it. The squint would, however, become a design constraint for any future configuration that interrogates the fluid from a distance rather than in the near field, and a cross-polarisation characterisation of the E-plane is correspondingly identified as future work in Section 4.

## 3. Transmission-Fluid Degradation Measurement: Measurement Model, Methodology and Results

### 3.1. Measurement Model, Measurand and Measurement Chain

Following the VIM [33], the measurement problem is stated explicitly before the experimental methodology. The quantity observed is the complex reflection coefficient Γ(f) of the antenna, referred to the SMA reference plane defined by the SOLT calibration; its logarithmic magnitude is denoted S_11_ (dB). When the antenna aperture is placed above the fluid cell, the observed quantity becomes the loaded reflection coefficient of a layered structure: the radiating aperture, a near-zero air gap g, a fluid layer of thickness t ≈ 9–10 mm, and the steel bottom of the reservoir, which acts as a reflective (short-circuit-like) backing. The cell is therefore a metal-backed fluid layer—a configuration analogous to the metal-backed sample geometry of free-space reflection methods for dielectric characterization—interrogated here through the near field of the array aperture. Conceptually, the measurement function is S_11_(f) = F[ε*(f); t, g, T, G], where ε*(f) = ε′(f) − jε″(f) is the complex permittivity of the fluid (the property of interest), T is temperature, and G collects the fixed geometrical parameters of the cell and antenna.

In the present proof of concept, the inverse problem—retrieving ε*(f) from S_11_(f)—is deliberately not attempted, because it requires an accurate electromagnetic model of the full cell and independently characterized reference liquids. Instead, the method is operated as a reference-comparison (differential) measurement: the measurand is the pair of indicator quantities (f_n_, L_n_), the frequency and level of the fluid-loaded resonance notch of the antenna, and fluid condition is assessed through the differences Δf_n_ and ΔL_n_ between a fluid under test and the fresh reference fluid measured in the same cell with the same calibrated chain. The measurement chain comprises the antenna aperture, the SMA connector (calibration reference plane), the coaxial cable, and the SOLT-calibrated VNA. The dominant influence quantities are: the fluid layer thickness (fill volume), the antenna-to-fluid standoff, ambient temperature, positioning repeatability of the motorised carriage, calibration residuals at the reference plane, instrument noise and drift, and connector/cable repeatability. Their treatment—Type A evaluation from repeated acquisitions and Type B considerations from the calibration and instrument properties, in the sense of the GUM [32]—is presented in Section 3.6.

### 3.2. Physical Sensing Principle

#### 3.2.1. Reflection-Coefficient Model of the Metal-Backed Fluid Cell

The measurement cell is a fluid layer of thickness t backed by the steel reservoir base, which acts as a short circuit. Treating the layer as a short-circuited transmission-line section filled with a medium of complex permittivity ε* = ε′ − jε″, the propagation constant and intrinsic impedance are

γ = jω√(μ_0_ε_0_ε*), η = η_0_/√ε* (3)

and the impedance presented by the cell to the aperture is

Z_cell(f) = η tanh(γt)(4)

in which γ is the complex propagation constant defined in Equation (3). In the lossless limit γ = jβ and Equation (4) reduces to Z_cell = jη tan(βt), the familiar input impedance of a short-circuited transmission-line section; the hyperbolic form is retained here because the fluid is lossy and γ is therefore complex.

The aperture loaded by this cell presents a total input impedance at the SMA reference plane

Z_L_(f) = R_L_(f) + jX_L_(f) (5)

from which the measured reflection coefficient follows as

Γ(f) = [Z_L_(f) − Z_0_]/[Z_L_(f) + Z_0_] (6)

with magnitude

|Γ| = |R_L_ − Z_0_ + jX_L_|/|R_L_ + Z_0_ + jX_L_| (7)

Equation (7) makes explicit that the depth of the notch is governed jointly by the departure of R_L_ from Z_0_ and by the residual reactance X_L_ at the matching frequency. Evaluating the electrical thickness of the cell for t = 9–10 mm and ε′ ≈ 2.2 at 4.75 GHz gives βt ≈ 76–85°, i.e., the cell is operated just below the quarter-wave condition, where ∂|Z_cell|/∂ε′ is large. This provides the physical rationale for the sensitivity of the configuration.

#### 3.2.2. Mechanism Identified from the Measured Data

**Figure 7:**
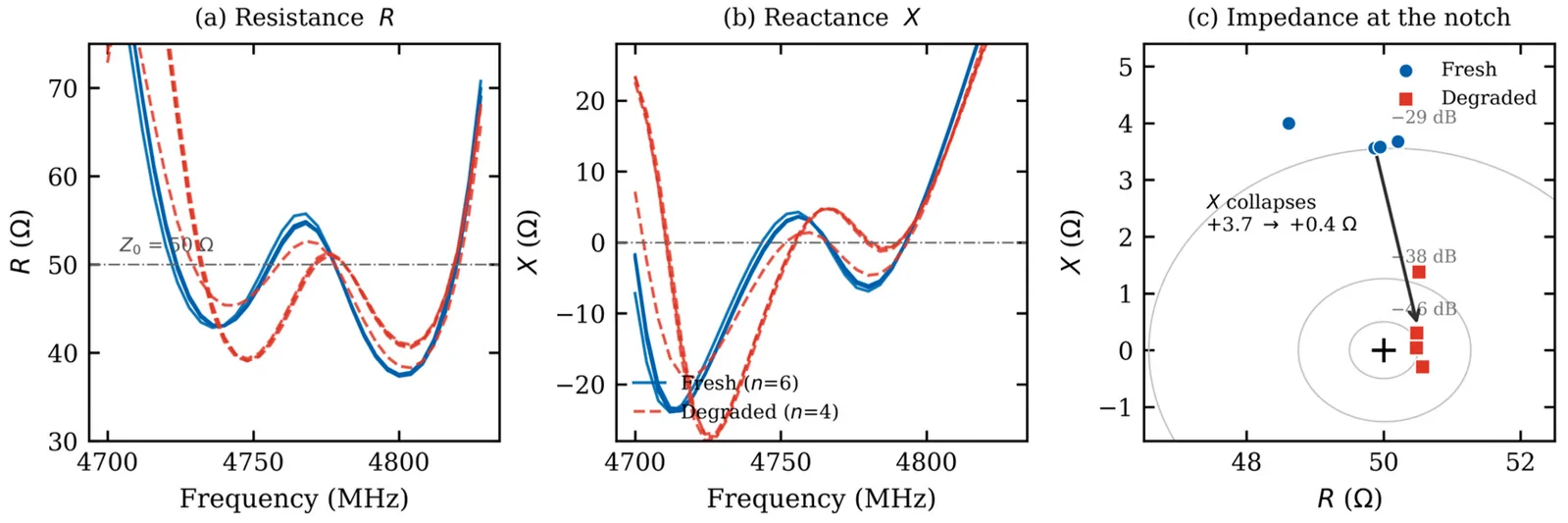
Decomposition of the measured loaded input impedance: (**a**) resistance R_L_(f); (**b**) reactance X_L_(f); (**c**) impedance at the notch for all ten acquisitions, with constant-|Γ| contours. The resistive part is essentially unchanged by degradation while the reactive part collapses from +3.7 Ω to +0.4 Ω, which accounts for both the upward frequency shift and the deepening of the notch.

Converting the measured complex reflection coefficient to the loaded input impedance at the notch (Figure 7) separates the two contributions in Equation (7). Averaged over the repeated acquisitions, the resistive part changes from 49.85 Ω (fresh) to 50.51 Ω (degraded), i.e., by +0.67 Ω, whereas the reactive part collapses from +3.69 Ω to +0.36 Ω, i.e., by −3.33 Ω. Substituting these values into Equation (7) reproduces the measured notch levels: |Γ| = 0.0370 (−28.6 dB) for the fresh fluid and |Γ| = 0.0062 (−44.1 dB) for the degraded fluid.

The mechanism is therefore reactive rather than resistive. Degradation shifts the reactance function XL(f) upward in frequency: the zero-reactance crossing moves by approximately +10 MHz, and at the new crossing the residual reactance is an order of magnitude smaller than for the fresh fluid. The notch consequently moves up in frequency and deepens for the same underlying reason. The two indicators are therefore not independent signatures but two observable consequences of a single reactive mechanism.

**Figure 8:**
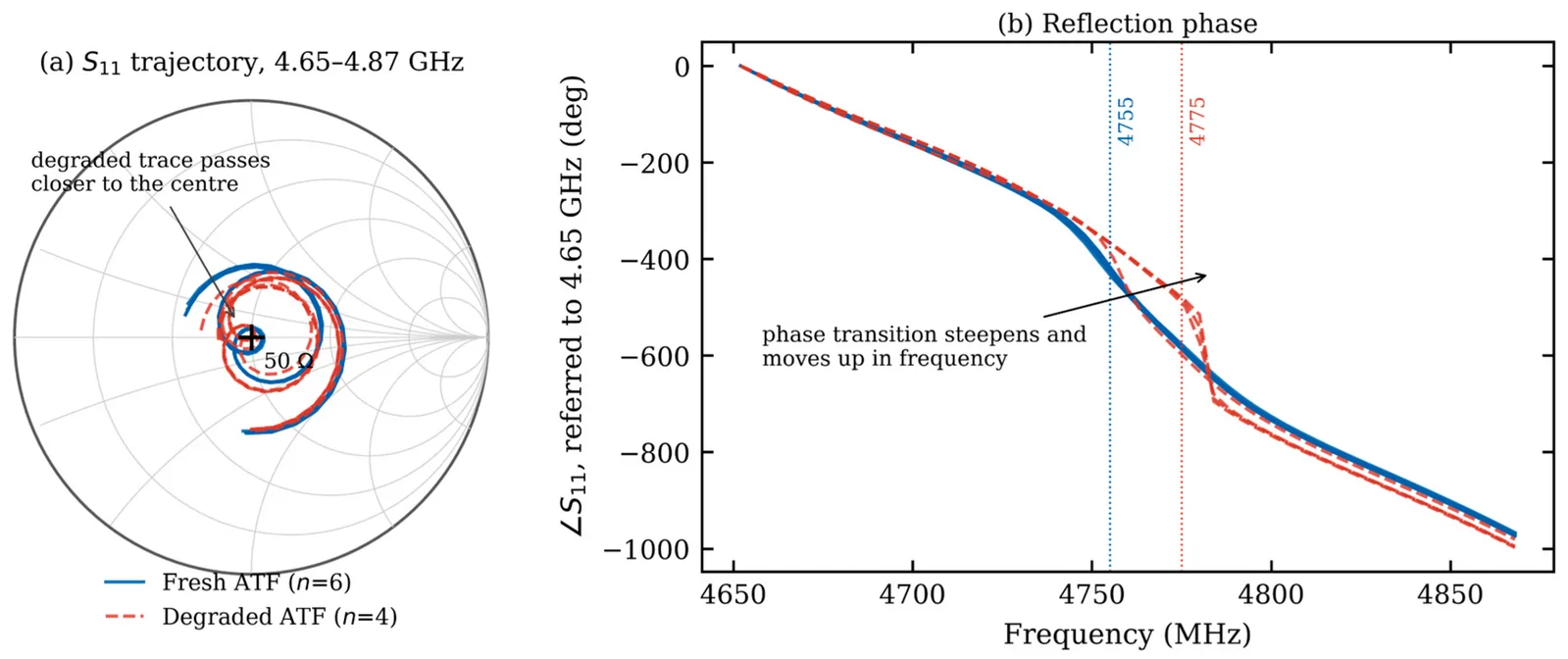
Complex reflection coefficient of the fluid-loaded antenna: (**a**) S_11_ trajectory on the Smith chart over 4.65–4.87 GHz, in which the degraded-fluid locus passes markedly closer to the 50 Ω centre; (**b**) reflection phase referred to 4.65 GHz, showing the steepening and upward movement of the phase transition. Fresh n = 6, degraded n = 4.

This is corroborated by the phase behaviour (Figure 8). The reflection-coefficient trajectory of the degraded fluid passes markedly closer to the 50 Ω centre of the Smith chart, the phase slope at the notch steepens from −7.7 deg/MHz to −9.6 deg/MHz, and the notch width evaluated 3 dB above the minimum narrows from 28 MHz to 10 MHz—all consistent with the reduced residual reactance.

It must be emphasised that the measurand responds to the bulk complex permittivity of the fluid. Thermal oxidation products, moisture ingress and metallic wear debris all raise ε′ and ε″ in the same direction, so a single-frequency reflection measurement of this type has no chemical selectivity and cannot attribute an observed change to a specific degradation pathway. Because the mechanism is a reactance shift observable across several loaded resonances, a multi-frequency feature vector combined with multivariate analysis is the appropriate route to pathway discrimination; this is identified as future work in Section 4.

A one-dimensional plane-wave evaluation of Equations (3)–(7) reproduces the sign and order of magnitude of the level change but does not reproduce the sign of the frequency shift. This is attributed to the limitations of a plane-wave treatment of what is a near-field, finite-aperture interaction; resolving it requires the full-wave simulation of the complete loaded cell identified in Section 4.

The real part of the relative permittivity of fresh mineral-base transmission fluids at microwave frequencies is typically in the range of 2.1–2.4 [8,13]; for the fresh fluid used in this study, ε′ ≈ 2.2 is adopted, consistent with published data for fresh mineral-base lubricating oils [8,13]. As the fluid degrades, both ε′ and ε″ increase, causing greater electromagnetic energy absorption and thereby modifying the loaded impedance seen by the antenna and hence both the frequency and the depth of the loaded resonance notch. Independent permittivity measurements of the fluid samples at microwave frequencies (e.g., by open-ended coaxial probe spectroscopy) were not performed in the present proof-of-concept study; such measurements are identified as a key priority in future work and would enable an explicit quantitative relationship between ε* and the observed S_11_ signature to be established. In the absence of a direct measurement, the assumed dielectric values are supported by multiple independent literature sources for fresh and aged mineral-base oils [8,11,12,13].

### 3.3. Fluid Samples

Both fluid samples are the same commercial automotive transmission fluid—identical brand and grade—so that formulation differences cannot confound the comparison. The fresh sample was drawn from a previously unopened container of the product; the degraded sample was drained from an in-service passenger vehicle during a scheduled fluid change at an automotive service workshop, after approximately 200,000 km of accumulated service. The service mileage is as reported by the workshop and is therefore approximate; the vehicle specification and its detailed service history were not documented, which is acknowledged as a limitation in Section 4. The exact commercial designation of the product was likewise not recorded; based on the transparent amber-yellow appearance of the fresh sample, a mineral-base formulation with a fresh-state ε′ in the 2.1–2.4 range typical of mineral-base oils [8,13] is assumed. The degraded sample had visibly darkened to a deep opaque dark-brown color and exhibited a faint burnt odor, both of which are well-documented indicators of advanced thermal oxidation and additive depletion in ATF [3,5]. No additional laboratory characterization of the fluid samples (such as TAN or kinematic viscosity) was performed in the present proof-of-concept study; such characterization, correlated with the RF measurement data, is identified as an essential component of future multi-point calibration work. Only these two extreme fluid conditions are compared in the present study; the implications of this binary comparison are discussed in Section 4.

The degradation state of the service sample is evidenced only by its documented service history (a scheduled fluid change at approximately 200,000 km, as reported by the workshop), its visual appearance and its odour. No independently measured chemical or physical quality parameter—total acid number, kinematic viscosity or particulate concentration—is available for either sample. The measurements reported here were performed in July 2025; the archived samples have since aged in storage, so a characterisation performed now would describe the samples in their present state rather than in the state in which they were interrogated. Such ground truth is therefore not recoverable retrospectively for this dataset and requires a new campaign in which the RF and laboratory measurements are performed contemporaneously. Accordingly, this study establishes that two ATF specimens of the same brand and grade and of markedly different service history produce reproducibly different and separable S_11_ spectra; it does not establish a relationship between the measured indicators and fluid degradation chemistry.

### 3.4. Measurement Setup, Instrumentation and Motorised Positioning System

**Figure 9:**
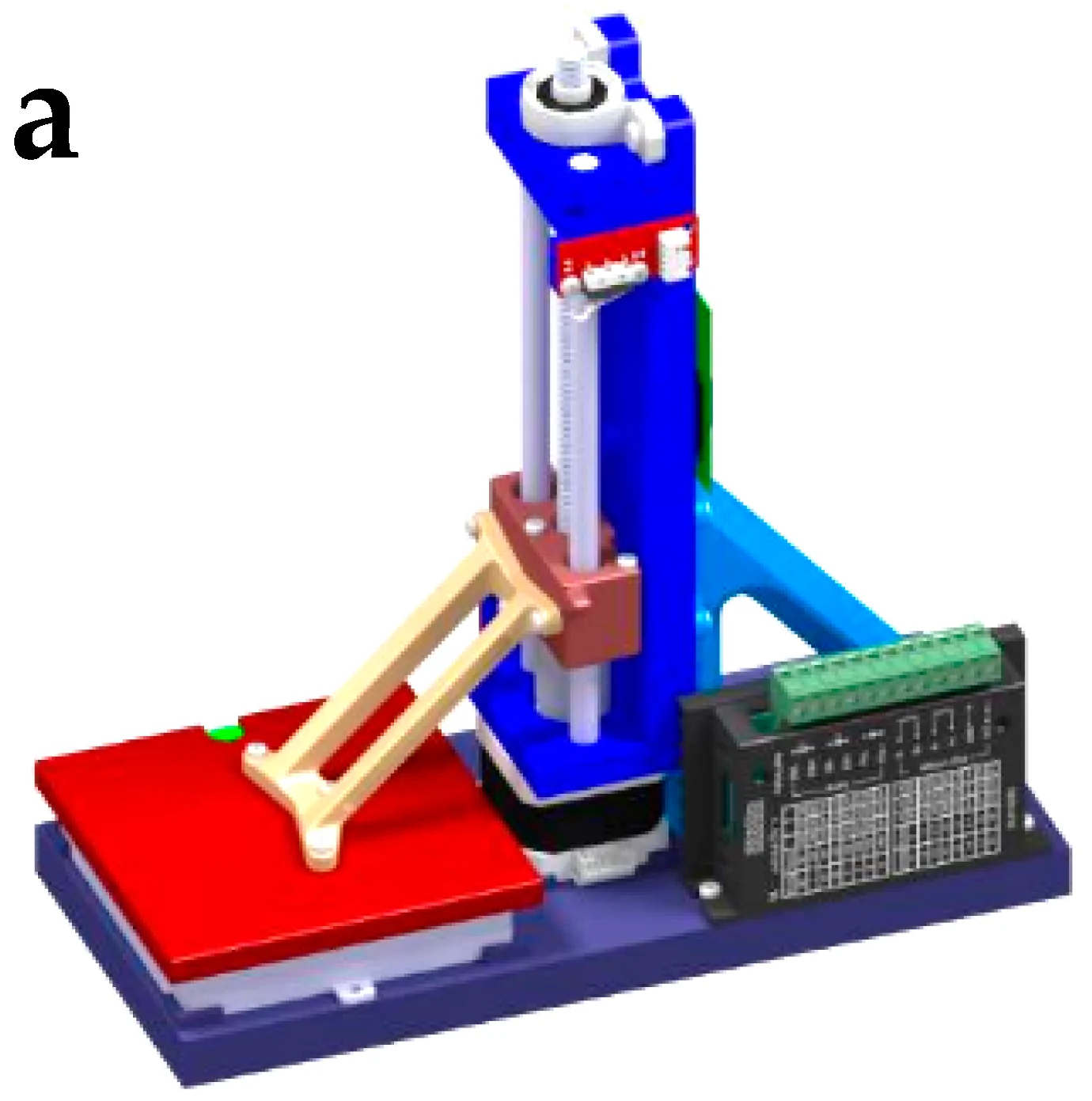

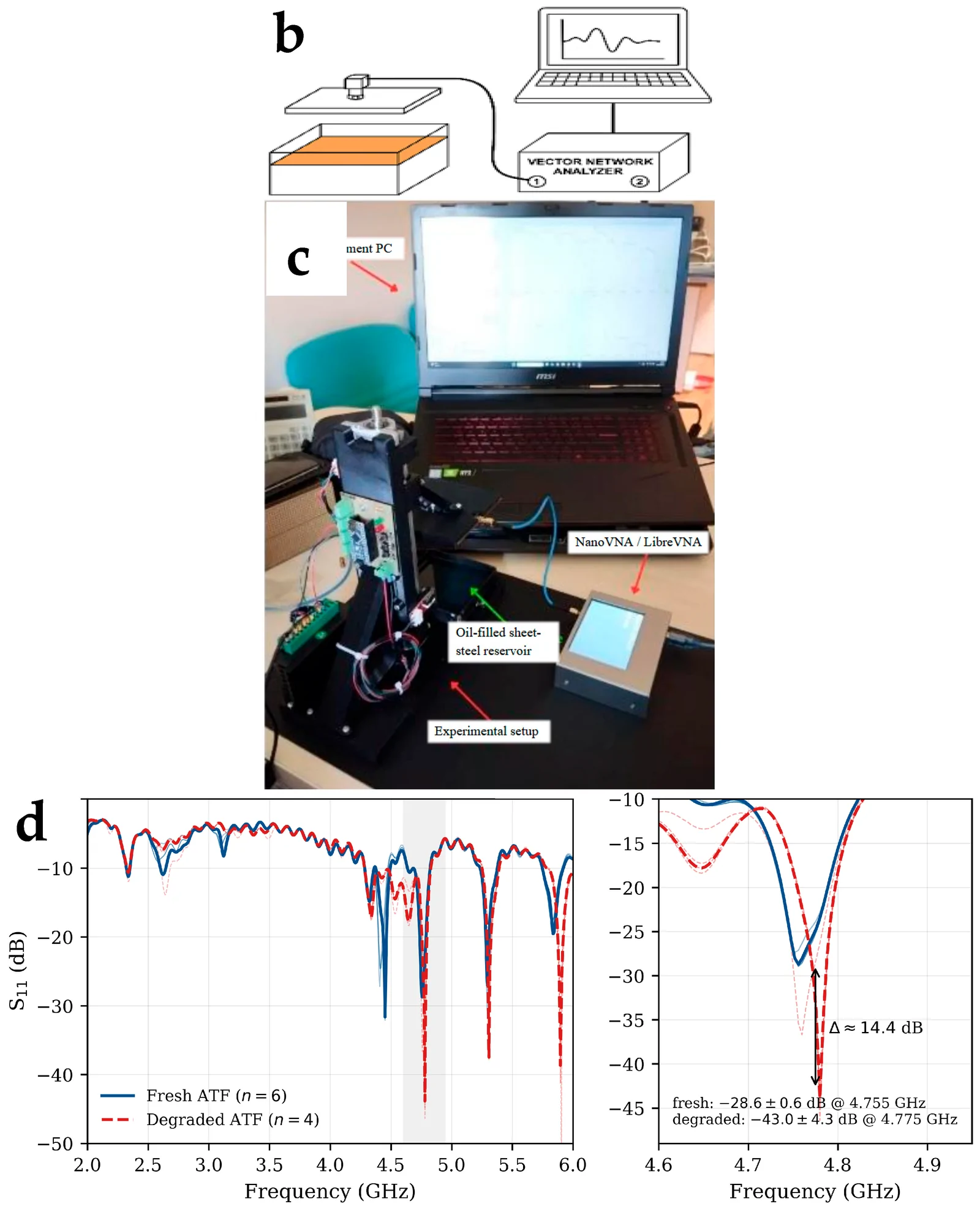
(**a**) CAD illustration of the proposed non-contact fluid measurement system; (**b**) schematic block diagram of the measurement setup showing the antenna, motorised positioning system, and VNA connection (SOLT-calibrated at the SMA reference plane); (**c**) photograph of the experimental configuration during the preliminary campaign (NanoVNA-class portable analyzer shown; the reported fluid measurements were acquired with the LibreVNA using the same mechanical system and reservoir, Section 3.4); (**d**) measured S_11_ spectra comparing fresh (unused) and degraded (~200,000 km) transmission fluid samples: (**left**) all repeated acquisitions (n = 6 fresh, n = 4 degraded) with representative traces emphasized; (**right**) detail of the loaded resonance notch with group statistics (mean ± standard deviation).

The measurement concept is illustrated in Figure 9a (CAD, computer-aided design), Figure 9b (schematic block diagram), and Figure 9c (photograph of the experimental configuration during the preliminary campaign).

A rectangular laser-cut sheet-steel reservoir with internal dimensions of 0.10 × 0.10 × 0.02 m (length × width × depth) was filled with approximately 90 ml90 mL of fluid, yielding a fluid layer thickness of ≈9–10 mm. The steel bottom of the reservoir acts as the reflective backing of the layered measurement cell defined in Section 3.1. To ensure repeatable, position-consistent measurements, the antenna was mounted on a custom-built motorised positioning system comprising a NEMA 17 stepper motor, USA; driving a trapezoidal lead screw guided by two linear bearings along fixed shafts, controlled via a TB6600 stepper motor driver, Japan; and an Arduino Nano microcontroller, USA. Upper and lower limit switches define the travel range and prevent mechanical overrun. Both fluid samples were measured at the same reference position defined by the lower limit switch, ensuring that the observed S11 signature is attributable primarily to differences in fluid dielectric properties and not to geometric variation. At this reference position the radiating (patch) face of the antenna is parallel to and directly above the fluid surface at a near-zero standoff (below approximately 2 mm); the copper surface does not contact the fluid, so the measurement remains non-contact and the fluid is neither penetrated nor contaminated. A systematic standoff-sensitivity map is nevertheless identified as future work in Section 4.

The reservoir has internal dimensions of 100 mm × 100 mm, so a nominal 90 mL fill corresponds to a mean layer thickness of 9.0 mm; the 9–10 mm range quoted reflects the uncertainty in fill volume and in the flatness of the laser-cut base. The measurement position is defined mechanically by a lower limit switch, which is the same physical stop for every acquisition, so the position is reproducible to the repeatability of the switch and stage rather than being set by operator judgement. Parallelism between the radiating face and the fluid surface is established by the fixed geometry of the carriage relative to the reservoir mounting plate, which is bolted to the same base. Fluid contact with the copper face did not occur in any reported acquisition; should it occur, the measurement would no longer be non-contact, the cell geometry of Section 3.1 would no longer apply, and the acquisition would be discarded and the antenna cleaned before continuing.

The standoff itself was not measured. The carriage was driven down to the limit switch and the antenna face was verified visually to be clear of the fluid surface; the resulting gap is estimated to be below approximately 2 mm but was not determined with a gauge, and no tolerance can therefore be quoted. We record this as a deficiency of the present campaign rather than offer an estimate that the data do not support. Because the limit switch is a single fixed mechanical stop, the standoff was nevertheless the same for every acquisition to the repeatability of the stage, which is the property the comparison relies on; its absolute value is required for the quantitative model and is listed in Table 2 as an uncharacterised influence quantity and in Section 4 as required future work.

**Figure 10:**
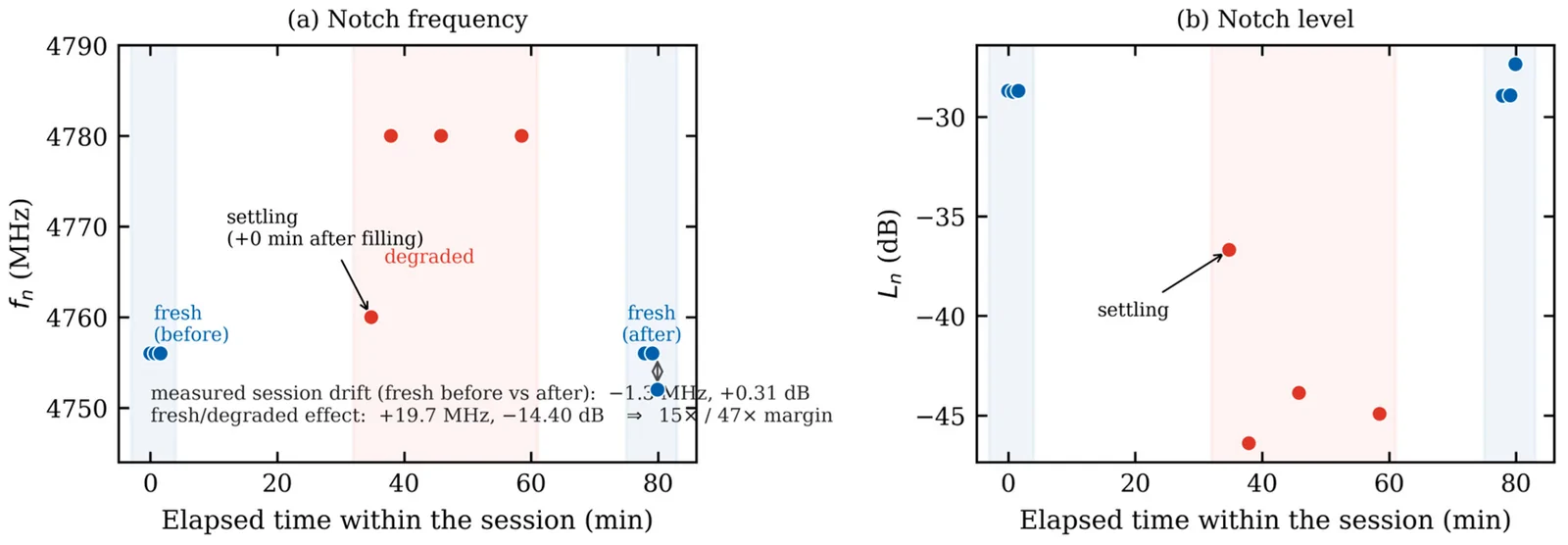
Bracketed acquisition sequence within the single 80 min session: (**a**) notch frequency; (**b**) notch level. The fresh fluid was measured both before and after the degraded block, giving a measured session drift of −1.3 MHz and +0.31 dB against a fresh/degraded effect of +19.7 MHz and −14.40 dB. The first degraded acquisition, recorded immediately after filling, shows the settling transient discussed in the text.

All ten fluid acquisitions were recorded on 7 July 2025 within a single session of 80 min, under one SOLT calibration, with the coaxial connection to the antenna undisturbed throughout. The sequence was bracketed: three fresh-fluid acquisitions at the start (elapsed 0, 1 and 2 min), four degraded-fluid acquisitions (35, 38, 46 and 59 min), and three further fresh-fluid acquisitions at the end (78, 79 and 80 min). Measuring the fresh fluid both before and after the degraded block provides a direct empirical measurement of the total drift of the measurement chain over the session, including calibration drift, connector and cable effects, and any thermal component. This is quantified in Section 3.6 and presented in Figure 10. The 35 MHz session-to-session variation reported in Section 2.3 refers to the unloaded antenna bench characterisation, which was performed in two separate sessions with the cable assembly disconnected and re-made between them; it does not apply to the fluid measurements reported here.

Two measurement campaigns were carried out with this fixture. Preliminary trials used a NanoVNA-class portable analyzer (usable to ≈4 GHz); the photograph in Figure 9c was taken during this preliminary campaign. All fluid results reported in this paper were subsequently acquired with a LibreVNA two-port vector network analyzer (100 kHz–6 GHz; LibreVNA-GUI v1.6.3). A full SOLT calibration was performed with the manufacturer’s calibration kit at the SMA reference plane, and the calibration was cross-checked by repeating it with an independent (PicoVNA) calibration kit, which produced consistent results. S_11_ spectra were recorded across 2–6 GHz with 1001 points (4 MHz frequency step).

### 3.5. Results and Discussion

The comparative S11 spectra, presented in Figure 9d, reveal a clear and systematic distinction between the electromagnetic signatures of the two fluid samples. Across the repeated acquisitions, the fresh fluid produces a loaded resonance notch of −28.6 ± 0.6 dB at 4.755 GHz (standard deviation of the notch frequency ±2 MHz; n = 6), whereas the degraded fluid produces a notch of −43.0 ± 4.3 dB at 4.775 GHz (±10 MHz; n = 4), where the quoted dispersions are Type A standard deviations of the repeats. Degradation therefore manifests in two observable indicators, shown in Section 3.2 to arise from a single reactive mechanism: the loaded notch shifts upward by ≈20 MHz and simultaneously deepens by ≈14.4 dB (difference in group means). Even in the worst case across all ten acquisitions—the shallowest degraded-fluid notch (−36.7 dB) against the deepest fresh-fluid notch (−28.9 dB)—the two specimens remain separated by ≥7.7 dB, i.e., the class separation between the two specimens exceeds the observed repeat dispersion by a wide margin. This behaviour is physically consistent with the elevated complex permittivity and loss tangent of aged oil [8,11,12], which modify the loaded aperture admittance and thereby both the matching frequency and the matching depth of the fluid-loaded antenna. Across the broader 2–6 GHz band, a consistent systematic difference between the fresh- and degraded-fluid spectra is observed at the other loaded resonances as well (e.g., near 4.45, 5.30 and 5.90 GHz, Figure 9d), confirming that the response is not confined to a single narrow resonance. The repeated spectra are mutually consistent: the median spread (maximum minus minimum across repeats, evaluated per frequency point) is ≈0.3 dB across the band for both fluids, rising in the immediate vicinity of sharp resonance notches (90th percentile 1.8 dB fresh/2.6 dB degraded), where the depth of a deep minimum is intrinsically sensitive to minute impedance changes. These repeatability data are the Type A input to the uncertainty discussion of Section 3.6.

Formal statistical tests are reported for both indicators. Given the small group sizes the non-parametric Mann–Whitney U test is taken as the primary analysis, with Welch’s *t*-test and Cohen’s d reported alongside it. For the notch level: U-test *p* = 0.0095, t = 6.63, *p* = 0.0064, d = 5.4. For the notch frequency: U-test *p* = 0.0080, t = 3.90, *p* = 0.0281, d = 3.1. Both indicators separate the two specimens at *p* < 0.05 by both tests. These tests compare two specimens and not two fluid populations; the *p*-values therefore quantify the reliability of the measured separation for this sample pair and do not generalise to ATF as a class, which would require multiple independent samples.

Quantitative figures of merit are collected in Table 3. The level and frequency sensitivities are reported as endpoint slopes, derived from two fluid conditions only: 0.072 dB and 100 kHz per 1000 km of accumulated service respectively, the latter corresponding to 0.42% of f_0_ over the full range. The 3σ resolutions referred to the fresh reference group are 1.8 dB (≈25,000 km equivalent) and 6.0 MHz (≈60,000 km equivalent). It is essential to emphasise that these are endpoint slopes and not calibration curves: they are derived from two extreme data points, they assume a linear relationship that transmission-fluid degradation is not expected to follow, and they must not be used for maintenance scheduling without the multi-point calibration described in Section 4.

### 3.6. Repeatability and Measurement Uncertainty Considerations

Table 2 summarizes the principal uncertainty contributions of the method in the sense of the GUM [32]. The Type A (statistical) contributions are evaluated directly from the repeated acquisitions of Section 3.5. The Type B contributions are assessed from the properties of the measurement chain: SOLT calibration residuals at the SMA reference plane (bounded in practice by the consistency obtained when the calibration was repeated with an independent calibration kit), the 4 MHz frequency grid (which quantizes the notch-frequency estimate), instrument noise and drift of the low-cost VNA (whose dynamic range decreases toward 6 GHz), and the mechanical reproducibility of the limit-switch-defined antenna position. Ambient temperature was not actively controlled and is listed as an influence quantity; all repeats of a given fluid were acquired under nominally identical laboratory conditions.

Two observations follow from Table 2. First, away from sharp resonance notches the repeat spread is small (median ≈ 0.3 dB), so the broadband spectral signature is highly reproducible. Second, the largest dispersion appears exactly where the indicator is read—the depth of a deep notch is intrinsically sensitive to small impedance perturbations (as also observed between sessions for the unloaded antenna, Section 2.3)—and this is properly reflected in the ±4.3 dB Type A dispersion of the degraded-fluid notch level. The essential metrological statement of this proof of concept is that the separation between the two specimens examined here between fresh and degraded fluid (14.4 dB between group means; ≥7.7 dB worst case over all repeats) exceeds the combined observed dispersion by a wide margin, so the binary classification is robust; converting the method into a quantitative degradation measurement will additionally require the multi-point calibration and independent fluid characterization identified in Section 4.

Third, the bracketed acquisition sequence described in Section 3.4 permits the total session drift to be measured directly. The fresh-fluid group acquired before the degraded block gives 4756.0 MHz and −28.72 dB; the group acquired after it gives 4754.7 MHz and −28.41 dB. The measured drift over the 80 min session is therefore −1.3 MHz and +0.31 dB, against a fresh/degraded effect of +19.7 MHz and −14.40 dB—margins of 15× and 47× respectively. Because this bound is obtained by repeating the same fluid at the two ends of the session, it encompasses every drift mechanism acting over that interval, including the thermal one. Ambient temperature was not recorded during the campaign; a thermal drift large enough to account for the observed frequency shift would have had to occur entirely within the degraded block and reverse itself before the closing fresh acquisitions, which is not plausible for a laboratory interior over this interval. Determining the temperature coefficient of the measurement in MHz/°C nevertheless remains necessary for any deployed implementation and is identified in Section 4.

Fourth, the first degraded acquisition, recorded immediately after the reservoir was filled, differs systematically from the three that follow: 4760 MHz and −36.69 dB at +0 min, against 4780 MHz and −46.40, −43.87 and −44.92 dB at +3, +11 and +24 min. The response stabilises within approximately three minutes of filling, consistent with levelling of the fluid surface, escape of entrained air and thermal equilibration of the fluid with the cell. Almost the entire dispersion of the degraded group (±10 MHz, ±4.3 dB) originates in this single acquisition; the three settled acquisitions give 4780 ± 0 MHz and −45.06 ± 1.27 dB. This acquisition has not been excluded and all reported statistics use the complete dataset of ten acquisitions. Fluid settling time after filling is instead entered in Table 2 as an influence quantity, and a minimum settling interval is specified as a protocol requirement for future campaigns.

## 4. Limitations and Future Directions

The comparison of only two extreme fluid conditions is insufficient to characterize the measurement response across intermediate degradation states or to validate the linearity assumption underlying the reported sensitivity metric. A multi-point calibration study using samples collected at regular service intervals (e.g., every 20,000 km), correlated with independently measured fluid quality parameters (TAN, viscosity index, particulate concentration), is essential. The fluid documentation of the present study is likewise limited: the commercial designation of the ATF and the detailed service history of the donor vehicle were not recorded, and future campaigns will use fully specified fluids with documented histories.

A full-wave simulation of the complete loaded cell—antenna, air gap, 9–10 mm fluid layer and steel backing—with a parametric sweep over ε′ and tan δ is the immediate next step for this work. Such a model is required to test whether the measurement model reproduces the sign and magnitude of both observed indicators, and in particular to resolve the frequency-shift sign that the one-dimensional treatment of Section 3.2 does not capture. Independent permittivity measurements of the fluid samples, by open-ended coaxial probe, are required alongside it to establish the quantitative link between ε* and the observed reflection signature. The next campaign should acquire samples at regular service intervals with RF and laboratory characterisation (total acid number, kinematic viscosity, particulate concentration) performed contemporaneously, and should record ambient temperature throughout so that the MHz/°C coefficient of the configuration can be determined.

The standoff distance was fixed at a near-zero, limit-switch-defined value in the reported campaign; the sensitivity of the response to standoff was not characterised, a systematic map of the S_11_ signature as a function of standoff (and of fluid layer thickness) is required to complete the sensitivity analysis of the measurement model. Direct permittivity measurements of the fluid samples at microwave frequencies—using an open-ended coaxial probe—are needed to establish an explicit quantitative link between ε* variation and the observed S_11_ signature, and to correlate the observed S_11_ shift with the known permittivity contrast between fresh and degraded oils [8,11,14].

A rigorous experimental comparison between a single-element patch sensor and the 16-element array under identical fluid conditions is required to quantify the spatial-averaging advantage of the array. In practical transmission systems, oil replacement is typically incomplete, resulting in a mixture of residual and fresh fluid; the measurement response as a function of mixing ratio (0–100% degraded) must therefore be characterized. The experimental protocol should be extended to include fluids contaminated with water [6], metallic wear particles, clutch debris, or exhibiting chemical degradation at different stages.

Regarding practical integration, the electromagnetic environment within a metallic transmission assembly—including shielding effects and electromagnetic interference (EMI) from actuators and control electronics—must be assessed. Machine learning algorithms trained on frequency-domain S11 feature vectors could enhance discrimination capability and enable automated fluid condition grading, consistent with emerging trends in microwave sensing for liquid characterization [14,15]; such tools would be employed on top of—not instead of—the calibrated measurement model developed here. Temperature-dependent characterization is also warranted. Finally, integration with a compact VNA module and wireless data transmission would constitute a significant step toward a field-deployable real-time ATF monitoring system.

## 5. Conclusions

This study has formulated and experimentally characterized a non-contact reflection-coefficient measurement method for assessing the degradation state of automotive transmission fluid in which a steel-backed layered fluid cell is interrogated through the S_11_ response of a 4 × 4 hexagonal patch array antenna and fluid condition is read from the frequency and level of the fluid-loaded resonance notch.

First, the fabricated antenna demonstrated well-matched performance between CST simulation and laboratory measurement: resonant frequencies of 4.852 GHz (simulated, S_11_ = −13.88 dB) and 4.725 GHz (measured, S_11_ = −24.32 dB); −10 dB bandwidths of ≈115 MHz (simulated) and ≈140 MHz (measured) around the design resonance; and a measured half-power beamwidth of 29.22° (relative pattern; no absolute gain calibration was performed). A closed-form cavity-model estimate of the element resonance (≈4.97 GHz) agrees with the full-wave result within ≈2.4%, providing the physical design rationale.

Second, for the single fresh/degraded sample pair examined here, repeated acquisitions (n = 6 fresh, n = 4 degraded) established that degradation shifts the loaded notch upward by ≈20 MHz and deepens it by ≈14.4 dB, with a worst-case separation of ≥7.7 dB across all repeats. Decomposition of the measured complex reflection coefficient shows that both effects arise from a single reactive mechanism: the resistive part of the loaded impedance at the notch is essentially unchanged (49.9 → 50.5 Ω) while the reactive part collapses (+3.7 → +0.4 Ω). The two specimens separate at *p* = 0.0095 (level) and *p* = 0.0080 (frequency) by the Mann–Whitney U test, against a bracketed session drift of −1.3 MHz and +0.31 dB. These repeats characterise the repeatability of the measurement on two specimens; they do not characterise fresh and degraded ATF as fluid classes, which would require multiple independent samples.

Third, the measurement chain was operated with an explicitly defined reference plane (SOLT calibration at the SMA connector, cross-checked with an independent calibration kit) and a limit-switch-defined antenna position, and the method was characterized metrologically: the median repeat spread is ≈0.3 dB across the band, the Type A dispersions of the indicator quantities are quantified, and the dominant influence quantities are identified in an explicit uncertainty budget (Section 3.6).

Fourth, the non-contact measurement configuration—combined with the broad-aperture array design matched to the reservoir footprint—offers practical advantages over contact-based and immersion-based methods: the fluid is neither penetrated nor contaminated, the aperture spatially averages the dielectric response over the full cell footprint, and the hardware cost remains low, rendering the proposed approach a promising candidate for condition-based maintenance strategies in automotive drivetrain systems. From a measurement-science perspective, the contribution of this work is the formulation of ATF condition assessment as an explicit reflection-coefficient measurement problem—with a defined measurand, reference plane, repeatability data and uncertainty considerations—rather than as a sensor-response demonstration alone; the reported repeat statistics provide the baseline against which future calibrated implementations of the method can be compared.

## Figures and Tables

**Table 1:** Comparison of representative RF, microwave, and antenna-based sensing approaches for liquid characterization. ATF = Automotive Transmission Fluid.

Ref.	Sensor/Method	Freq. Band	Target Fluid	Key Sensing Metric	Non-Contact?	ATF?
[18] Zhu et al. (2020)	Microfluidic microstrip patch antenna	~2.2 GHz	Lubricating oil (moisture)	≥20 MHz resonance shift	No (microfluidic)	No
[19] Rajendran et al. (2020)	Self-complementary dipole antenna	1.1–3.3 GHz	Food/beverage liquids	Resonance frequency shift	Partial	No
[20] Jun et al. (2019)	EBG structure + planar antenna	GHz range	Alcohols (generic)	S11/reflected-phase shift	Partial	No
[22] Vafanejad (2009)	Microacoustic MEMS resonator	Acoustic	Lubricating oil	Density & viscosity	No (immersed)	No
[23] Ikhmis et al. (2017)	Helical antenna sensor	~2 GHz	Generic liquids	Frequency shift	Yes	No
[24] Dalgaç et al. (2021)	Chiral metamaterial sensor	X-band (~10 GHz)	Car lubrication oil	Transmission-freq. shift	Yes	No
[25] Baghelani et al. (2021)	Planar microwave resonator	GHz range	Mineral oil (water contam.)	ppm-level Δf sensitivity	Yes	No
[26] Chuma et al. (2022)	Metamaterial resonator + magnets	GHz range	Oil (wear particles)	Particle detection	Partial	No
[27] Zhang et al. (2021)	Submersible microwave sensor	GHz range	Edible oil	S21 spectral features	No (submersed)	No
[14] Palandoken & Gocen (2025)	Planar microwave sensors (review)	Various	Various liquids	Frequency/phase variation	Yes	No
[15] Wu et al. (2024)	IDS-microstrip + RF circuits	GHz range	Binary aqueous solutions	+57.5–175.3% sensitivity vs. prior art	Yes	No
[28] Han et al. (2023)	Microfluidic microwave sensor, annular microstrip patch	GHz range	Lubricating oil	Resonance frequency shift	No (microfluidic)	No
[29] Zaarour et al. (2024)	Additively manufactured waveguide sensor	X-band	Engine oil	S-parameter spectral change	No (filled cell)	No
[30] Santhosh Kumar & Ganesh Madhan (2026)	Dielectric cavity antenna sensor	GHz range	Engine oils/liquid chemicals	Resonance frequency shift	No (contact cell)	No
This work	4 × 4 hexagonal patch array antenna, S11 measurement with repeatability & uncertainty characterization	2–6 GHz	Automotive transmission fluid (ATF)	Δf ≈ +20 MHz and ΔS11 ≈ 14.4 dB at the loaded notch (n = 10 repeats)	Yes	Yes

**Table 2:** Principal uncertainty contributions and influence quantities of the fluid measurement (indicator quantities: loaded notch frequency f_n_ and level L_n_).

Contribution/Influence Quantity	Type	Evaluation	Value/Observation
Repeatability of notch level L_n (fresh)	A	Std. dev. of n = 6 repeats	0.6 dB
Repeatability of notch level L_n (degraded)	A	Std. dev. of n = 4 repeats	4.3 dB (deep-notch sensitivity)
Repeatability of notch frequency f_n	A	Std. dev. of repeats	2 MHz (fresh)/10 MHz (degraded)
Broadband spectral repeatability	A	Per-frequency spread of repeats	median ≈ 0.3 dB; 90th percentile 1.8–2.6 dB
Calibration residuals at SMA plane	B	SOLT; cross-check with independent (PicoVNA) kit	consistent results between kits; residuals not separately quantified
Frequency-grid quantization	B	Sweep step	4 MHz (fluid sweeps); 25 MHz (antenna bench sweep)
Instrument noise/drift (LibreVNA)	B	Manufacturer specification; single-session operation	dynamic range decreases toward 6 GHz; not separately quantified
Antenna position (standoff) reproducibility	B	Limit-switch-defined reference position	Not measured; limit-switch stop identical for all acquisitions; absolute standoff uncharacterised
Fluid layer thickness/fill volume	B	Nominal 90 mL fill	Nominal 90 mL over 100 × 100 mm = 9.0 mm; a +/−0.5 mm by about +/−4.5 deg at 4.75 GHz, comparable to delta(eps prime) ~0.1 (analytical bound, Section 3.2)
Ambient temperature	B	Not actively controlled	Not recorded; total session drift bounded by bracketing at −1.3 MHz/+0.31 dB over 80 min (Section 3.6)
Fluid settling time after filling	A	Comparison of the first degraded acquisition with the three that follow	Response stabilises within about 3 min of filling; first acquisition differs by 20 MHz/8 dB; minimum settling interval now specified

**Table 3:** Quantitative figures of merit of the measurement, evaluated from the repeated acquisitions of the single fresh/degraded sample pair. The sensitivity entries are endpoint slopes derived from two fluid conditions and are not calibration curves.

Figure of Merit	Value	Basis
Level sensitivity, S_L	0.072 dB per 1000 km	Endpoint slope; 14.4 dB over ~200,000 km
Frequency sensitivity, S_f	100 kHz per 1000 km	Endpoint slope; 19.7 MHz over ~200,000 km
Normalised frequency sensitivity	0.42% of f0	Δf_n/f_n over the full range
Resolution, level channel (3σ)	1.8 dB ≈ 25,000 km equivalent	3 × σ of the fresh reference group
Resolution, frequency channel (3σ)	6.0 MHz ≈ 60,000 km equivalent	3 × σ of the fresh reference group
Discrimination ratio	26:1	Worst-case separation (7.7 dB)/median repeat spread (0.3 dB)
Notch width, 3 dB above minimum	28 MHz → 10 MHz	Fresh → degraded
Effect size, level channel	Cohen’s d = 5.4	Pooled standard deviation
Statistical separation	*p* = 0.0095 (level); *p* = 0.0080 (freq.)	Mann–Whitney U test
Session drift (bracketed)	−1.3 MHz; +0.31 dB	Fresh group before vs. after the degraded block
Selectivity	None (bulk permittivity response)	Responds to total ε*, not to a specific pathway

## Data Availability

The raw S-parameter (Touchstone) datasets and other data supporting the findings of this study are available from the corresponding author upon reasonable request.

## Abbreviations

The following abbreviations are used in this manuscript:

RF	Radio Frequency
S11	Reflection coefficient (scattering parameter)
CST	Computer Simulation Technology (CST Microwave Studio)
VNA	Vector Network Analyzer
EBG	Electromagnetic Bandgap
SMA	SubMiniature version A (connector type)
AUT	Antenna Under Test
ATF	Automotive Transmission Fluid
TAN	Total Acid Number
IoT	Internet of Things
FR4	Flame Retardant 4 (epoxy laminate substrate)
SOLT	Short-Open-Load-Thru (VNA calibration standard)
GUM	Guide to the Expression of Uncertainty in Measurement (JCGM 100)
VIM	International Vocabulary of Metrology (JCGM 200)
HPBW	Half-Power Beamwidth
CAD	Computer-Aided Design
EMI	Electromagnetic Interference

## Nomenclature

The following symbols are used in this manuscript:

Symbol	Definition	Unit
*Γ*	Complex reflection coefficient at the SMA reference plane	–
*S*_11_	Reflection coefficient expressed logarithmically	dB
*f*_n_	Frequency of the fluid-loaded resonance notch	MHz
*Δf*_n_	Change in notch frequency between fluid conditions	MHz
*L*_n_	Level of the fluid-loaded resonance notch	dB
*ΔL*_n_	Change in notch level between fluid conditions	dB
*f*_0_	Reference frequency used for normalised sensitivity	GHz
*f*_r_	Closed-form dominant-mode resonance, Equation (2)	GHz
*ε**	Complex relative permittivity, ε* = ε′ − jε″	–
*ε′*, *ε″*	Real and imaginary parts of the relative permittivity	–
*tan δ*	Dielectric loss tangent, ε″/ε′	–
*ε*_r_	Relative permittivity of the FR4 substrate	–
*ε*_eff_	Effective dielectric constant of the substrate	–
*η*, *η*_0_	Intrinsic impedance of the fluid and of free space	Ω
*γ*, *β*	Complex propagation constant and phase constant in the fluid	m^−1^
*λ*_0_	Free-space wavelength	mm
*λ*_g_	Guided wavelength in the microstrip medium	mm
*t*	Fluid layer thickness in the measurement cell	mm
*g*	Antenna-to-fluid standoff (air gap)	mm
*h*	Substrate thickness	mm
*a*	Side length of the regular hexagonal patch	mm
*a*_eq_	Equivalent circular-patch radius, Equation (1)	mm
*a*_e_	Effective circular-patch radius including fringing	mm
*D*	Aperture dimension used in the far-field criterion 2D^2^/λ	m
*Z*_L_	Loaded input impedance at the reference plane	Ω
*R*_L_	Resistive part of the loaded input impedance	Ω
*X*_L_	Reactive part of the loaded input impedance	Ω
*Z*_0_	Reference (system) impedance, 50 Ω	Ω
*K*	Surface current density on the conductor layer	A/m
*θ*, *φ*	Elevation and azimuth angles of the far-field coordinate system	deg
*S*_L_	Level sensitivity (endpoint slope)	dB per 1000 km
*S*_f_	Frequency sensitivity (endpoint slope)	MHz per 1000 km
*T*	Fluid temperature	°C
*G*	Fixed geometrical parameters of the cell and antenna	–
*n*	Number of repeated acquisitions	–
*σ*	Standard deviation of a set of repeated acquisitions	dB or MHz
*d*	Cohen’s effect size	–
*p*	Significance level of a statistical test	–
